# Obesity-related kidney disease: Beyond hypertension and insulin-resistance

**DOI:** 10.3389/fendo.2022.1095211

**Published:** 2023-01-16

**Authors:** Tarek Arabi, Areez Shafqat, Belal Nedal Sabbah, Nader Ashraf, Hassan Shah, Humzah Abdulkader, Adhil Razak, Ahmad Nedal Sabbah, Ziad Arabi

**Affiliations:** ^1^ College of Medicine, Alfaisal University, Riyadh, Saudi Arabia; ^2^ Division of Nephrology, Department of Medicine, King Abdulaziz Medical City, Riyadh, Saudi Arabia; ^3^ King Abdullah International Medical Research Center, Riyadh, Saudi Arabia; ^4^ College of Medicine, King Saud bin Abdulaziz University for Health Sciences, Riyadh, Saudi Arabia

**Keywords:** chronic kidney disease, obesity, cellular senescence, chronic inflammation, adipokines, senotherapies

## Abstract

Chronic kidney disease (CKD) causes considerable morbidity, mortality, and health expenditures worldwide. Obesity is a significant risk factor for CKD development, partially explained by the high prevalence of diabetes mellitus and hypertension in obese patients. However, adipocytes also possess potent endocrine functions, secreting a myriad of cytokines and adipokines that contribute to insulin resistance and induce a chronic low-grade inflammatory state thereby damaging the kidney. CKD development itself is associated with various metabolic alterations that exacerbate adipose tissue dysfunction and insulin resistance. This adipose-renal axis is a major focus of current research, given the rising incidence of CKD and obesity. Cellular senescence is a biologic hallmark of aging, and age is another significant risk factor for obesity and CKD. An elevated senescent cell burden in adipose tissue predicts renal dysfunction in animal models, and senotherapies may alleviate these phenotypes. In this review, we discuss the direct mechanisms by which adipose tissue contributes to CKD development, emphasizing the potential clinical importance of such pathways in augmenting the care of CKD.

## 1 Introduction

Obesity is a contributing risk factor of 20-25% of chronic kidney disease (CKD) cases worldwide (1). As per the 2011-2014 National Health and Nutrition Examination Survey, 44.1% of CKD patients in the United States of America (USA) were obese (2). The number of end-stage kidney disease (ESKD) kidney transplant recipients who were obese also grew by 44% from 1999—2009 (3). Diabetes and hypertension—the two most common causes of CKD worldwide—frequently accompany obesity and are often put forward as the major causes of obesity-related CKD. However, obesity is a risk factor CKD-related disability and mortality after adjusting for diabetes and hypertension (4, 5). Othman et al. demonstrated that non-diabetic obese patients were more likely to undergo CKD progression than non-obese subjects (6). These results suggest an independent mechanism by which obesity damages the kidney.

Although lifestyle changes, such as weight loss, are essential for managing obesity, most patients fail to achieve adequate or sustained weight loss (7). Recent clinical trials report that the glucagon-like-peptide-1 (GLP-1) receptor agonist semaglutide and the GLP and gastric inhibitory peptide (GIP) receptor agonist tirzepatide induce significant weight loss in obese patients; high dose terzepatide (10-15 mg weekly) achieved > 20% reductions in body weight, resembling that achieved after bariatric surgeries (8–12). Sattar et al. concluded that GLP-1 receptor agonists slowed decline in estimated glomerular filtration rate (eGFR), ameliorated progression to ESKD, and reduced kidney disease-related mortality (13). Bariatric surgeries are an option for morbidly obese patients who cannot lose weight and are refractory to anti-obesity medications. Bariatric surgery reduces systemic inflammation, proteinuria, and glomerular hyperfiltration in obese CKD patients (14, 15). Bariatric surgery also decreases the 5-year risk of mortality by 79% in obese pre-dialysis CKD patients (16). Such data demonstrate that decreasing adiposity betters various indices of kidney function and mitigates CKD development and progression.

The management of obesity-related CKD is still in its infancy, and evidence-based guidelines are yet to be established (1). Improvements in risk stratification and management protocols are urgently needed to improve the care of obese-related CKD. While diabetes and hypertension are significant contributors to obesity-related CKD, recent decades of research have shown that adipocytes are potent endocrine cells, releasing adipokines which exert direct pathologic effects on the kidney (17). Adipokines also indirectly damage the kidney by contributing to the development of insulin resistance and hypertension (18).

Alternatively, CKD induces several endocrine and immunologic dysregulations in adipose tissue. Identifying the key players of this adipose-renal axis may have clinical practice-changing implications, given the strong links between obesity and CKD and their paralleled rise in prevalence. Identifying mediators of adipose tissue-induced kidney disease is essential in improving risk prediction models of CKD in obese patients and identifying targets for pharmacotherapies. This review discusses the adipose tissue-derived mediators of CKD and translational research on how such mechanisms are targeted.

## 2 Adipose tissue inflammation in obesity and chronic kidney disease

Chronic low-grade inflammation is a biological hallmark of aging—termed inflammaging (19). Obesity promotes inflammaging, explaining why obese individuals experience age-related chronic disease prematurely (20, 21). Conversely, limiting fat development or inducing adipose tissue depletion extends health and life span (22). Both obesity and aging impair adipogenesis, the process by which adipocyte progenitors differentiate into functional, insulin-responsive adipocytes (23). Consequently, adipose tissue cannot buffer circulating lipids, which then deposit ectopically in other organs, such as the liver, skeletal muscle, and kidney, causing lipotoxicity. Lipotoxicity impairs insulin signaling in the kidney, liver, and skeletal muscle, causing insulin resistance (24).

Individual adipocytes hypertrophy in response to impaired adipogenesis (25). Hypertrophic adipocytes promote adipose tissue inflammation by producing tumor necrosis factor-α (TNF-α) and interleukin-6 (IL-6) (26). These proinflammatory cytokines are critical to the onset of insulin resistance; mice lacking TNF-α have lower circulating free fatty-acids and are protected from insulin resistance (27). Hypertrophic adipocytes also produce macrophage chemoattractant protein-1 (MCP-1), recruiting adipose tissue macrophages (ATMs) (28). ATMs constitute less than 10% of the total cell population of adipose tissue in lean individuals and mice but increase disproportionately in obesity to make up 40-50% of the adipose tissue cellular compartment (28). Indeed, increased ATM recruitment is histologically evident, revealing ATMs surrounding dead or dying adipocytes, forming crown-like structures and engulfing lipid droplets (29). ATMs in obesity are polarized towards a proinflammatory M1 phenotype, elaborating proinflammatory cytokines such as TNF-α (30). Therefore, hypertrophic adipocytes and M1-polarized ATMs actively contribute to adipose tissue inflammation and insulin resistance. In agreement with these findings, knocking out MCP-1 or its receptor attenuates macrophage infiltration into adipose tissue and reduces insulin resistance (31). Pharmacologically polarizing ATMs towards an M2 phenotype also reduces adipose tissue inflammation in high-fat diet (HFD) obese mouse models (32, 33).

The array of cytokines and signaling molecules released by adipose tissue renders them capable of modulating the inflammatory and immunologic phenotypes of various organs, including the kidney. In this light, adipose tissue inflammation exerts detrimental effects on renal function. Plasma TNF-α and IL-6 are elevated in obese pateints and are associated with CKD incidence and severity independent of diabetes (34, 35). Weight loss or bariatric surgery normalizes these proinflammatory cytokines and reduces glomerular hyperfiltration (14). IL-1β is another pro-inflammatory cytokine elevated in obesity. Importantly, patients with sustained IL-1β elevations post-bariatric surgery experienced no improvement in hyperfiltration (36).

Adipose tissue fibrosis is another important mediator of adipose tissue inflammation in obesity (37). In this regard, ATMs secrete matrix metalloproteinase-14 (MMP-14) to induce extracellular matrix remodeling by activating MMP-2 and MMP-9 (38). Furthermore, certain MMPs impair adipogenesis in obesity (38). In support of the contribution of MMPs to adipose tissue inflammation, MMP-12-deficient mice fed a high-fat diet (HFD) showed better insulin sensitivity and adipogenesis and an anti-inflammatory M2 ATM phenotype compared to wild-type mice fed an HFD (39). MMP-12 depletion also attenuated glomerular inflammation and renal fibrosis (39), indicating that changes in the inflammatory and immune phenotypes of adipose tissue affect the kidney. Along this line, pharmacologically polarizing ATMs to an M2 phenotype has renoprotective effects by preventing glomerular and mesangial expansion and fibrosis (32, 33).

Hypoxia contributes to adipose tissue inflammation and fibrosis. Rapid adipocyte hypertrophy in obesity outgrows its blood supply, resulting in hypoxia, cell death, and inflammation (40). Adipocyte tissue hypoxia activates hypoxia-inducible factor-1α (HIF-1α). HIF-1α does not elicit pro-angiogenic responses in adipose tissue but rather pro-fibrotic and pro-inflammatory transcriptional programs, leading to fibrosis and inflammation (37, 41). Inhibiting HIF-1α *via* PX-478 or introducing a dominant negative mutation prevents these fibrotic and inflammatory responses, even under a high-fat challenge (42). Hypoxic conditions in visceral adipose tissue downregulate the insulin receptor, which is reversible if oxygen supply is restored. Hypoxia-related insulin insensitivity in adipose tissue is mediated by micro-RNA 128, which destabilizes mRNA encoding the insulin receptor (43).

Therefore, adipose tissue inflammation in obesity is multi-factorial and drives renal dysfunction. This adipose-renal crosstalk is bidirectional. CKD reduces subcutaneous fat volume with a redistribution of fat to visceral depots and ectopic lipid deposition in skeletal muscle and the liver with consequent lipotoxicity (44). Ectopic lipid deposition also occurs in the kidneys in CKD, increasing renal inflammation (44). A recent study observed that exposing adipose tissue to uremic serum activates NFκB and HIF-1α, which drive adipose tissue inflammation. Indeed, adipose tissue sampled from dialysis patients also exhibits higher inflammatory markers (45), suggesting that it may be a source of the chronic low-grade inflammation observed in CKD patients in a manner unrelated to excess adiposity (46). CKD promotes macrophage infiltration into adipose tissue and consequent inflammation, leading to glucose intolerance and insulin resistance (44, 47, 48). Martos-Rus et al. recently demonstrated significantly higher ATM density in the adipose tissue of ESKD subjects than BMI-matched controls (49). ATM recruitment in CKD may be IL-6-dependent, as IL-6-KO mice showed reduced ATM densities comparable to wild-type mice. Uremic serum also directly activates ATMs to a pro-inflammatory M1 phenotype (49). Lastly, uremia alters the adipokine profile of adipocytes. Incubating human adipocytes with uremic serum increases leptin secretion and lipolysis while decreasing perilipin mRNA transcripts—perilipin promotes fat storage as triglycerides in adipose tissue (50–52). Urea accumulation in CKD also increases oxidative stress in adipose tissue, leading to the production of adipokines resistin and retinol-binding protein-4, which contribute to insulin resistance (48).

## 3 Adipokine alterations in obesity and effects on the kidney

**Figure 1:**
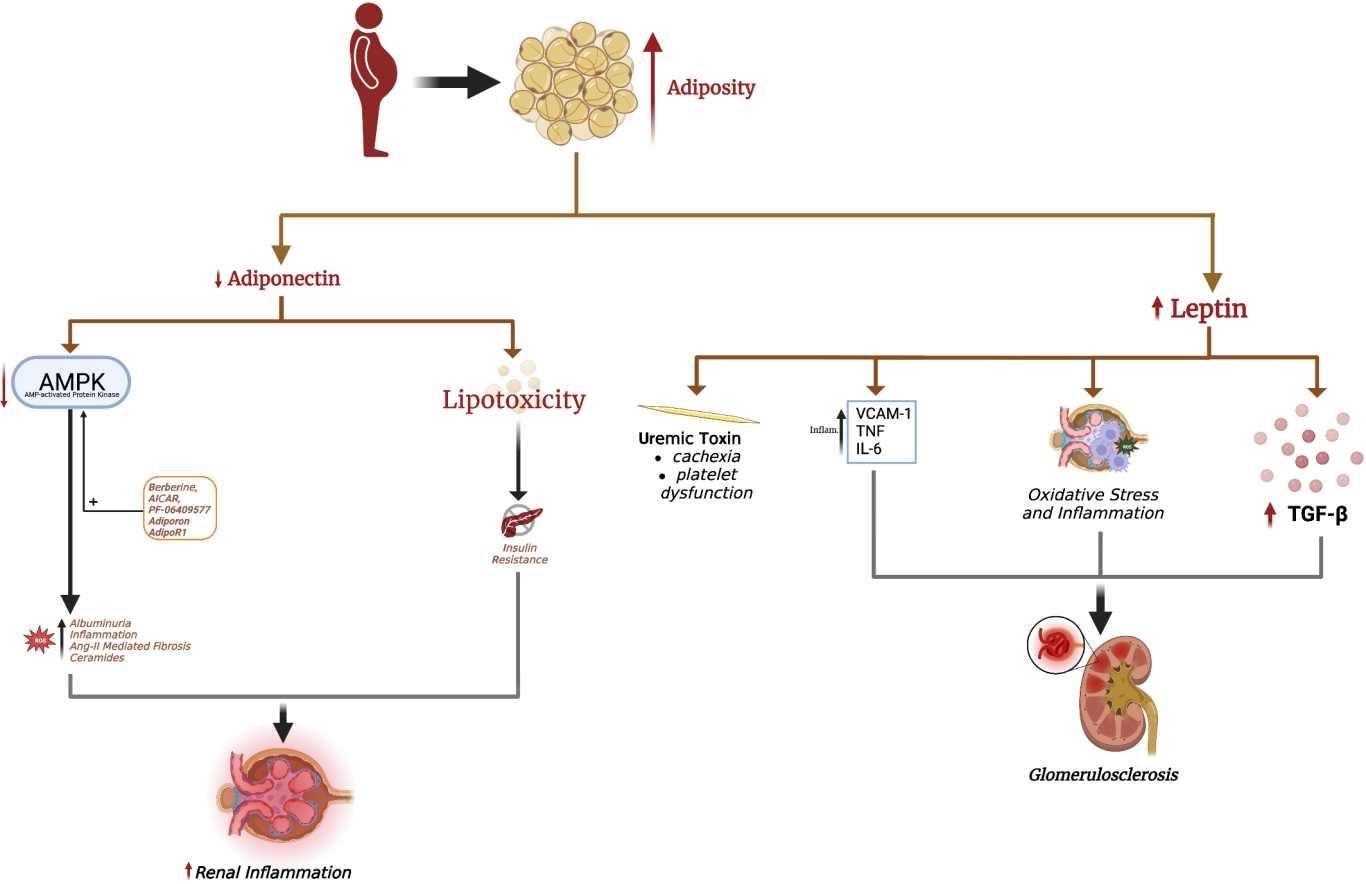
Excess adiposity increases serum leptin and decreases adiponectin. Hyperleptinemia promotes renal fibrosis, leading to glomerulosclerosis seen in obesity-induced glomerulopathy. Leptin is also considered a uremic toxin, contributing to uremic cachexia and platelet dysfunction. The decrease in renal adiponectin effect in obesity is causal in the development of albuminuria. Loss of adiponectin effect in obesity also increases renal oxidative stress, fibrosis, and inflammation. Adiponectin-mediated AMPK activation is responsible for many of adiponectin's renoprotective effects, with a decrease in AMPK implicated in albuminuria and renal injury. The adiponectin receptor agonist AdipoRon and direct AMPK activators AICAR, berberine, and PF-06409577 are pharmacological strategies to increase renal AMPK levels and mitigate obesity-related kidney disease.

Adipocytes produce various adipokines, enabling them to modulate the function of remote organs, such as the kidney. Below we discuss the most studied adipokines, leptin and adiponectin, and how alterations in these adipokines contribute to obesity-related CKD (**Figure 1**). Conversely, CKD also changes serum leptin and adiponectin levels, which may contribute to CKD stage progression and systemic complications.

### 3.1 Leptin

In conditions of nutrient excess, adipocytes produce leptin to modulate CNS activity, promote satiety, and increase energy expenditure. Obesity is associated with hyperleptinemia and leptin resistance (53, 54). Indeed, leptin levels are 5-10x higher in obese patients compared to non-obese individuals (55, 56). Since the kidney is the primary organ responsible for leptin clearance (57), CKD is also associated with hyperleptinemia, the degree of which correlates with the CKD stage (18, 58, 59). Park et al. recently demonstrated a significant correlation between elevated serum leptin levels and the risk of CKD in men after adjusting for eGFR and age (60). Such associations are even more evident in females, probably owing to sex-specific differences in circulating leptin levels (61).

Numerous studies have shown leptin to induce glomerulosclerosis and hypertension, both risk factors to CKD (62). The short form of the leptin receptor (Lep-Ra) is the predominant leptin receptor expressed in the kidney compared to the long form (Lep-Rb). Glomerulosclerosis and renal fibrosis in obese mice have been linked to Lep-Rb-dependent JAK2-STAT signaling in renal mesangial cells (63). Leptin promotes TGFβ-1 release and type IV collagen and fibronectin production in the glomerulus, leading to proteinuria and glomerulosclerosis (62). This effect was initially found to be mediated *via* adenosine monophosphate-activated protein kinase **(**AMPK) activation (64), which paradoxically inhibits TGFβ-1 and protects against renal fibrosis in several mouse models (65). This discrepancy suggested that leptin-mediated fibrosis may additionally involve other signaling pathways in the kidney. Indeed, activation of the p38/MAPK signaling pathway is involved in leptin-mediated renal fibrosis (64). Leptin also induces endothelial dysfunction (ED) by upregulating vascular adhesion molecules such as intercellular adhesion molecule-1 (ICAM-1) and vascular cell adhesion molecule-1 (VCAM-1) through AKT/GSK3β and Wnt/β-catenin signaling pathways, promoting renal inflammation and vascular remodeling (66, 67). Lastly, leptin promotes oxidative stress in renal tubular epithelial cells and stimulates monocytes to release IL-6 and TNF-α, promoting renal inflammation (68, 69).

Leptin is also considered a uremic toxin, contributing to many CKD complications including cachexia, protein-energy wasting (PEW), insulin resistance, hypertension, cardiovascular disease, and bone pathologies (70). PEW in CKD features anorexia, increased energy expenditure, decreased protein stores and muscle mass, and weight loss. Leptin suppresses food intake and increases energy expenditure through binding mineralocortin-4 receptors (MC4-R) in the hypothalamus, leading to uremic cachexia (71–73). Inhibiting MC4-R improves cachexia and reduces skeletal muscle wasting in preclinical models, but this needs further investigation in humans (73).

Leptin increases the risk of cardiovascular disease, the most common cause of mortality in CKD patients. Leptin induces hypertension by increasing sympathetic outflow, decreasing nitric oxide (NO) production, and increasing endothelin-1 in endothelial cells (74–76). Leptin promotes atherogenesis through endothelial cells, macrophages, and smooth muscle cells *via* the Lep-Rb receptor, which is reviewed elsewhere (77). Similarly, leptin binding to the Lep-Rb receptor on platelets enhances ADP signaling to induce platelet aggregation, which may cause the platelet dysfunction characteristic of uremia (78, 79). Lastly, hyperleptinemia decreases glucose-stimulated insulin release from pancreatic β-cells and impairs insulin signaling in hepatocytes (80, 81), although it should be noted that normal leptin levels enhance insulin release (80, 81).

Therefore, obesity-associated hyperleptinemia may contribute to renal pathology and CKD, mainly by causing secondary glomerulosclerosis. Furthermore, CKD-associated hyperleptinemia may contribute to numerous CKD complications. In agreement with these findings, leptin-deficient mice are significantly protected against albuminuria, glomerular crescent formation, macrophage infiltration, and glomerular thrombosis (82). Inhibiting leptin using specific antibodies or antagonists also substantially reduces blood pressure in mice with diet-induced obesity (83) and alleviates cachexia in CKD mice (84). Weight loss, either through lifestyle interventions, pharmacotherapies, or bariatric surgeries, is associated with significant decreases in leptin levels (85–89), but whether leptin normalization after weight loss directly improves renal function remains to be investigated.

### 3.2 Adiponectin

Adiponectin secretion is decreased in obesity, promoting the development of obesity-related chronic complications. The development of adiponectin-KO animal models have allowed causal relationships to be drawn between adiponectin deficiency and several aspects of the metabolic syndrome. For example, adiponectin-KO mice develop hepatic steatosis, which is attenuated by transfecting the adiponectin gene (90, 91). In skeletal muscle, adiponectin stimulates beta-oxidation and reduces lipid deposition and consequent lipotoxicity (92). Furthermore, adiponectin inhibits lipolysis and stimulates triglyceride storage in subcutaneous adipose tissue. Adiponectin, therefore, promotes fat storage in AT and increases insulin sensitivity, with its decrease in obesity a causal factor in insulin resistance, lipotoxicity, and metabolic syndrome manifestations (92).

The renoprotective effects of adiponectin are well-documented (18). Two adiponectin receptor isoforms, ADIPOR1 and ADIPOR2, are expressed in the kidney. Stimulation of ADIPOR1 and ADIPOR2 activate AMPK and peroxisome-proliferator activated receptor-α (PPAR-α), respectively, which attenuate renal inflammation, fibrosis, glomerulosclerosis, podocyte effacement, and albuminuria (17). A rise in intracellular AMPK by ADIPOR1 activation in podocytes inhibits NADPH oxidase and reduces permeability to albumin (93). In this light, non-obese non-diabetic mice who are adiponectin-deficient still develop effacement of podocyte foot processes and albuminuria due to increased oxidative stress (93, 94). In mesangial cells, adiponectin increases AMPK to attenuate angiotensin-II-induced TGF-β1 production, decreasing renal fibrosis (95).

Adiponectin also exerts anti-inflammatory effects on the kidney. For example, MCP-1 binds to its cognate CCR2 receptor to promote macrophage infiltration into the kidneys and renal inflammation (96). Adiponectin-deficient CKD mice develop significant albuminuria, tubulointerstitial fibrosis, and inflammation characterized by high MCP-1, TNF-α, NADPH oxidase, and VCAM-1 upregulation (97). Adiponectin administration *via* an adenovirus vector significantly reduces albuminuria, tubulointerstitial fibrosis, glomerular hypertrophy, and inflammation by lowering TNF-α, NADPH oxidase, and VCAM1 (97). Adiponectin has also been shown to directly stimulate IL-10 production by macrophages and decrease IL-6 and TNF-α, suggesting polarization to an M2 phenotype (98).

Lastly, ceramides are a group of sphingolipids implicated in renal disease. Serum levels of several ceramides are independent risk factors for CKD development and stage progression (99), as well as insulin resistance and lipotoxicity (100). Ceramides also act at several levels of the insulin signal transduction pathway to impair insulin signaling. Notably, both ADIPOR1 and ADIPOR2 possess intrinsic basal ceramidase activity, which is enhanced by adiponectin binding (101). Elevated ceramidase activity by ADIPOR1 and ADIPOR2 overexpression increases insulin sensitivity and glucose utilization while opposing hepatic steatosis (102). Ceramidase metabolizes ceramides into sphingosine-1-phosphate, which has anti-apoptotic effects and may even induce proliferation (103). These studies indicate that the pleiotropic metabolic, anti-apoptotic, and insulin-sensitizing effects of adiponectin may at least partly involve amplifying receptor-associated ceramidase activity.

Serum adiponectin is lower in obese patients compared to lean individuals. ADIPOR1 and ADIPOR2 expression is also reduced in the kidneys of obese and diabetic mice (104). Therefore, kidneys from obese mice and humans showed reduced AMPK levels (105). Treatment with 5-aminoimidazole-4-carboxamide-1-β-D-furanoside (AICAR), which enhances adiponectin-mediated AMPK signaling, increases AMPK levels in obese kidneys and reduces mesangial expansion and albuminuria (106). The antioxidant resveratrol also restores ADIPOR expression in the kidney and increases AMPK activation in diabetic mice, associated with reductions in albuminuria, oxidative stress, and inflammation (104). The molecule berberine enhances adiponectin signaling through AMPK to ameliorate renal pathology in diabetic mice (107). In animal models of diabetic nephropathy and obesity, the AMPK agonist PF-06409577 and adiponectin receptor agonist AdipoRon reduce proteinuria, inflammation, and renal fibrosis (108–110). These results suggest that targeting adiponectin receptors or AMPK directly may be beneficial in obesity- and diabetes-related kidney disease.

Despite adiponectin having numerous renoprotective effects, adiponectin levels are paradoxically increased in CKD and are positively correlated with albuminuria, CKD stage, and mortality, independent of body mass index (BMI) (58, 111). Adiponectin also predicts adverse cardiovascular outcomes in CKD patients (112). Unlike leptin, higher adiponectin levels in CKD cannot be simply explained by decreased renal clearance because the liver clears the high-molecular-weight form of adiponectin (113). Therefore, why adiponectin is elevated in CKD and is predictive of disease severity remains investigational.

Tian et al. induced CKD in non-obese mouse models with deoxycorticosterone acetate-salt (DOCA) and angiotensin II infusion (114). Transgenic adiponectin-overexpressing CKD mice showed significantly lower albuminuria, glomerular and interstitial fibrosis, and attenuated effacement of podocyte foot processes. Markers of tubular injury and inflammation were also lower in the transgenic models (114). These results are contrary to the unfavorable prognostic effect attributed to adiponectin in CKD patients. Yang et al. demonstrated that elevated adiponectin levels were associated with the presence of bone marrow-derived fibroblasts in kidneys with unilateral ureteral obstruction and ischemia/reperfusion injury (115). Adiponectin-deficient mice showed reduced renal fibroblast and M2 pro-fibrotic macrophage infiltration. The same study also showed adiponectin to activate AMPK on bone-marrow-derived monocytes, thereby increasing α-smooth muscle antigen (α-SMA) and production of extracellular matrix proteins. Therefore, the Yang et al. study suggested inhibiting the adiponectin/AMPK axis may ameliorate fibrotic renal disease (115). Similarly, Perri et al. reported that administration of lipopolysaccharide (LPS) induces adiponectin production by renal tubular epithelial cells to cause renal fibrosis (116). Numerous other studies have demonstrated the production of adiponectin by the kidney itself (117, 118). However, how kidney-derived adiponectin contributes to circulating adiponectin levels and any potential functional differences are not yet known.

PPAR-α is also known to exert renoprotective effects. Boor et al. demonstrated PPAR-α expression in the renal tubular epithelium but not the interstitium. PPAR-α levels decreased after fibrosis induction through unilateral ureteral obstruction and 5/6 nephrectomy (119). Treatment with the PPAR-α agonist BAY PP1 significantly increased PPAR-α expression, correlated with a reduction in tubulointerstitial fibrosis, inhibition of interstitial fibroblasts, lower TGF-β1 levels, and slowed down the progression of renal dysfunction. Therefore, PPAR-α in tubular epithelial cells attenuates fibrosis upon renal injury (119).

## 4 Cellular senescence in obesity and CKD

Cellular senescence was initially described by Hayflick and Moorhead when they observed that fibroblasts stop dividing after a set number of cell divisions (120). This cell cycle arrest was due to telomere attrition. The list of senescence-inducing stimuli has exponentially grown, but most culminate in DNA damage or oncogene activation, which activate senescence-inducing pathways. Hence, senescence is defined as an irreversible growth arrest upon the cell’s exposure to DNA-damaging or mitogenic stimuli. Senescence is characterized by numerous structural, biochemical, and metabolic alterations: a flattened and enlarged cellular morphology, decreased nuclear Lamin B1 expression, increased p53, p16^INK4a^ and/or p21^CIP1^ expression, elevated mitochondrial ROS production, elevated senescence-associated lysosomal β-galactosidase (SA-β gal) activity, apoptosis resistance *via* upregulation of senescence-associated anti-apoptotic pathways (SCAPs), and elaboration of a senescence-associated secretory phenotype (SASP) (121). The transient induction of senescence is considered physiological and critical to embryogenesis, wound healing, and tumor suppression. However, the chronic accumulation of senescent cells is implicated in the pathogenesis of numerous age-related disorders, including osteoporosis, obesity, stroke, neurodegenerative diseases, CKD, cancer, myocardial infarction and the geriatric syndromes (frailty, sarcopenia, and mild cognitive impairment) (122). Cellular senescence is indeed considered a biological hallmark of aging.

### 4.1 Cellular senescence in adipose tissue

Senescence plays a crucial role in propagating age-related diseases (123). Senescent cells accumulate in most tissues and organs with aging, including in adipose tissue. Importantly, obesity increases the senescent cell burden in adipose tissue. The p53-dependent DNA damage response is the main inducer of senescence in adipose tissue (124). A study showed that DNA polymerase-η *KO* mice (to increase DNA damage) accumulate senescent cells in adipose tissue (125). SREBP1—a transcription factor involved in regulating the expression of genes encoding proteins involved in lipid metabolism—was recently found to also facilitate DNA repair in adipocytes (126). Deletion of SREBP1 increased DNA damage and accelerated senescence in adipocytes, followed by adipose tissue inflammation and consequent insulin resistance (126). Mice with senescent cell accumulation in adipose tissue are more prone to obesity and adipose tissue inflammation, even with a standard chow diet (125).

**Figure 2:**
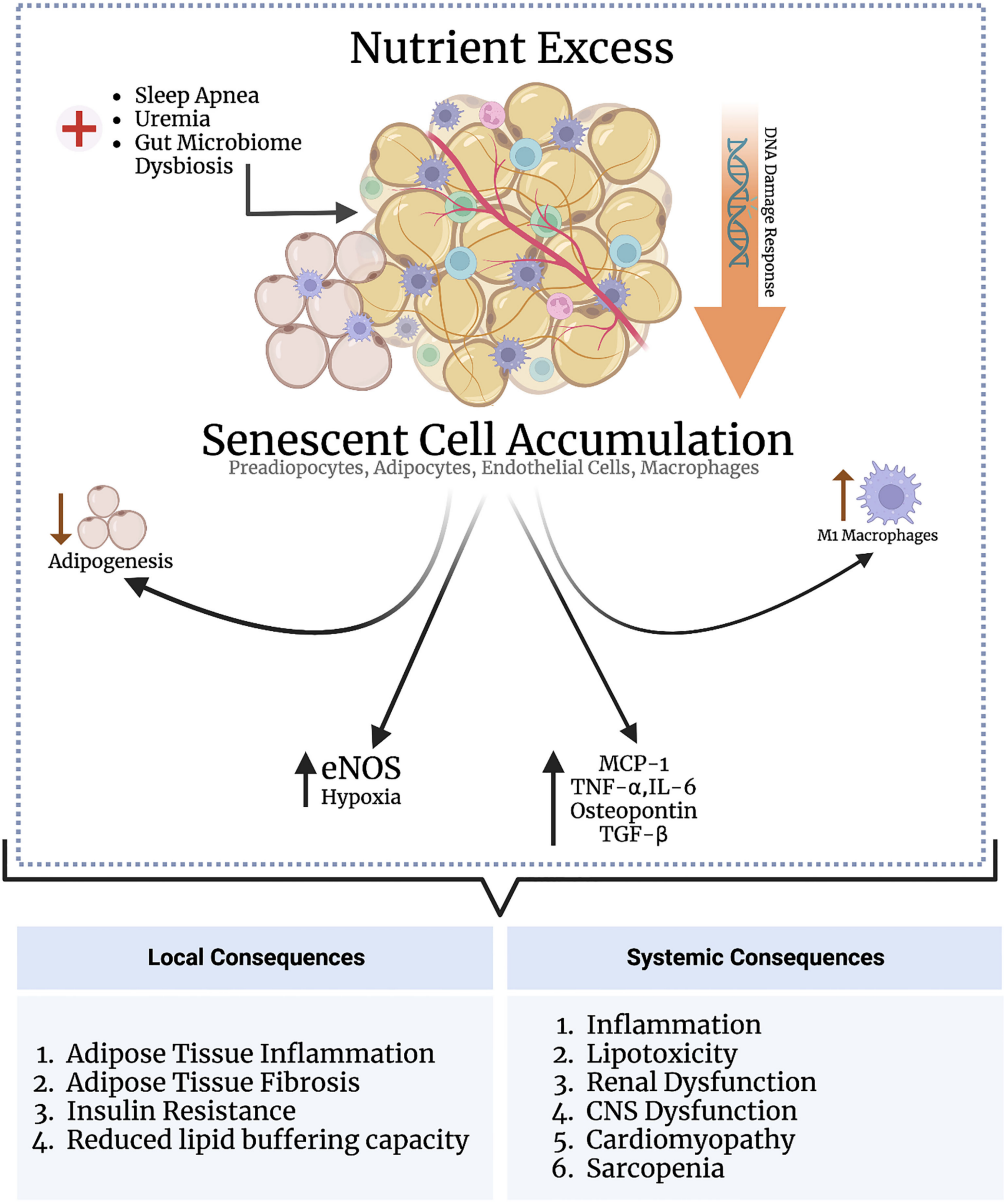
Nutrient excess triggers senescence in adipose tissue through DNA damage response signaling. Various other conditions, such as sleep apnea, uremia, and gut microbiome dysbiosis, may directly indce senescence in the adipose tissue independent of nutrient status. The adipose tissue is populated by various subsets of cells, including preadipocytes, adipocytes, endothelial cells, and macrophages. Senescence induction in preadipocytes impairs adipogenesis; senescence in macrophages and adipocytes enhances adipose tissue inflammation; and senescence in andothelial cells promotes adipose tissue hypoxia, inflammation, and fibrosis. An increased senescent cell burden in adipose tissue exerts several systemic consequences through the SASP as part of the inflammaging process, including cardiomyopathy, cognitive dysfunction, and renal impairment.

Oxidative stress-induced senescence in adipose tissue is linked to higher leptin, IL-6, and TNF-α production in the SASP, suggesting that adipocyte senescence may be causal in obesity-related chronic inflammation (124, 127). Depleting senescent cells in adipose tissue improves glucose homeostasis and insulin resistance (discussed below). Activin A is another SASP component which disrupts insulin signaling by decreasing the expression of insulin-dependent transcription factors including PPARγ and CCAAT-enhancer-binding protein α (C/EBPα) (128). These observations suggest that senescence in adipose tissue results in the production of cytokines and chemokines, leading to adipose tissue inflammation and insulin resistance. Adipose tissue is composed of many different cell types, each exhibiting varying susceptibility to senescence. The discussion herein focuses on the major cell types comprising adipose tissue and the causes and consequences of senescence induction in these cell types (**Figure 2**).

#### 4.1.1 Preadipocytes

Preadipocytes are adipocyte precursors are responsible for fat cell turnover by replacing dead and dying cells—i.e., adipogenesis. Both irradiation and telomere attrition induce senescence in preadipocytes, thereby impairing adipogenesis. Impaired adipogenesis in obesity in turn drives hypertrophic expansion of the subcutaneous fat compartment, increasing adipose tissue inflammation and metabolic dysfunction (129). Senescent preadipocytes also secrete a proinflammatory SASP, driving adipose tissue macrophage infiltration and inflammation. Accordingly, eliminating senescent preadipocytes in obese mice reduces adipose tissue inflammation and improves insulin sensitivity (130). These results indicate that senescence in preadipocytes contributes to adipose tissue inflammation and insulin resistance by impairing adipogenesis.

#### 4.1.2 Endothelial Cells

The vasculature of adipose tissue is not fenestrated. Transport across the vasculature into the adipose tissue interstitium is mediated by ECs, which express key transport proteins such as CD36 and fatty acid binding proteins (FABPs) that transport fatty acids between adipose tissue and blood (131). Importantly, PPARγ activation in ECs enhances transendothelial lipid transport and fat storage in adipose tissue. Free fatty acids activate PPARγ. Additionally, activated endothelial cells release PPARγ ligands that activate PPARγ in adipocytes (132). Adipose tissue storage of fats, therefore, depends on normal endothelial cell function, and depleting PPARγ in endothelial cells leads to systemic hyperlipidemia (133). ECs also produce factors that regulate adipose tissue blood flow, such as NO and angiogenic factors that increase adipose vascularity.

Adipose tissue EC senescence can be detrimental to normal adipose tissue function. In this context, ECs of HFD-mice undergo numerous p53-dependent gene expression alterations, including endothelial nitric oxide synthase (eNOS) downregulation, a change associated with insulin resistance (134). However, this study did not explore if p53 expression was associated with other senescence-related cellular alterations. Along similar lines, Barinda et al. showed that senescent ECs release a SASP that propagates senescence in mature adipocytes in a paracrine manner, associated with downregulation of the insulin receptor on mature adipocytes and, consequently, reduced insulin sensitivity (135). Cellular senescence also reduces PPARγ activation, decreasing the ability of endothelial cells to transport fatty acids, and the fat storage capacity of adipocytes (136). Lastly, senescent ECs isolated from visceral adipose tissue of obese individuals show higher expression of hypoxia-related genes and elaborate a proinflammatory SASP (136). Therefore, EC senescence may impair the lipid-buffering capacity of adipocytes by reducing PPARγ and cause adipose tissue hypoxia and inflammation.

#### 4.1.3 Mature adipocytes

Mature adipocytes are not expected to enter the cell cycle and divide; they respond to obesity by hypertrophying rather than dividing. However, Li et al. recently demonstrated that mature adipocytes can enter the cell cycle and increase in cell number in response to obesity and hyperinsulinemia (137). Chronic hyperinsulinemia induces premature adipocyte senescence, which release a SASP comprising MCP-1, TNF-α and IL-6 that drives adipose tissue inflammation (125, 137). Mature adipocyte senescence in obesity occurs before adipose tissue inflammation and insulin resistance, suggesting a causal relationship between adipocyte senescence and these phenotypes (138).

#### 4.1.4 Macrophages

Senescence is associated with adipose tissue inflammation *via* the SASP. Although ATMs play a key role in adipose tissue inflammation and consequent insulin resistance, there is a paucity of data on whether they can senesce and what consequences this may have on the inflammatory phenotype of adipose tissue. An elevated senescent cell burden in adipose tissue with SASP expression drives ATM recruitment and polarization into a proinflammatory M1 phenotype (139). Notably, depleting macrophages attenuates adipose tissue inflammation and fibrosis, with improvements in glucose tolerance parameters, indicating that macrophage infiltration contributes to senescence-induced adipose tissue dysfunction. However, whether ATMs themselves senesce was not determined in this study (140). A recent study demonstrated that senescent macrophages accumulate in the visceral adipose tissue derived from obese subjects who underwent bariatric surgery, and their numbers correlated with BMI, insulin resistance and degree of hyperinsulinemia (141). Importantly, both senescent adipocytes and macrophages elaborated a pro-inflammatory SASP, which prompted the authors to suggest that premature adipose tissue senescence in obesity contributes to inflammaging, possibly explaining why obese individuals develop age-related disease prematurely (141).

#### 4.1.5 Systemic

HFD mouse models accumulate senescent cells in multiple organ systems, which is associated with functional impairment. HFD mice show senescent cell accumulation in the liver and hepatic steatosis (142). In the brain of HFD mice, senescent cells accumulate near the lateral ventricle, which is associated with anxiety and gliosis (143). Kidneys of HFD mice also reveal a higher senescent cell burden, associated with renal dysfunction (144). Sawaki et al. demonstrated that aging adipose tissue releases osteopontin and TGF-β—in the SASP—which stimulate cardiac fibroblasts and drive myocardial fibrosis (145). Removing visceral adipose tissue in these mice reduced cardiac fibroblast activation, increased fibroblast senescence, and ameliorated myocardial fibrosis (145). Although this study did not directly examine senescence in visceral adipose tissue, Khan et al. reported that an elevated senescent cell burden in adipose tissue is associated myocardial fibrosis. p53-KO mice or removing senescent adipose tissue mitigated myocardial fibrosis (146). To correlate these findings clinically, myocardial fibrosis is a known mediator of obesity-associated cardiomyopathy; clinical studies are needed to explore whether targeting senescence may be beneficial in ameliorating this condition (145).

### 4.2 Senescence in CKD

Numerous studies report a higher senescent cell burden in diseased and aged kidneys. The regenerative potential of the kidney after injuries diminishes with aging and CKD. Cellular senescence is believed to impair regenerative mechanisms in aged and diseased kidneys, leading to maladaptive repair and fibrosis.

Proximal tubular epithelial cells are particularly affected by senescence. Biopsies of transplanted kidneys and those with glomerular diseases stain positive for senescence markers p16^INK4a^ and p21^CIP1^ in the proximal tubular epithelial cells (147, 148). Telomere attrition, elevated SA-β gal, p16^INK4a^, and p21^CIP1^ expression have also been directly correlated with IgA nephropathy progression (149). Baker et al. showed that senescent cells accumulate in aging kidneys in INK-ATTAC transgenic mice. An elevated renal senescent cell burden was associated with glomerulosclerosis, which was attenuated by depleting senescent cells in INK-ATTAC mice (150). Braun et al. demonstrated that transplanted kidneys in wild-type mice exhibit elevated senescence markers and incrementally undergo atrophy and fibrosis (151). By contrast, transplanted kidneys in p16^INK4A^-KO mice show less atrophy and fibrosis after ischemia-reperfusion injury (151). Transplanting kidneys from senescence-depleted mice consistently show better longevity and proliferation of tubular epithelial cells. An elevated senescent cell burden may therefore contribute to long-term allograft kidney deterioration and CKD development in humans (151).

*In vivo* mouse models of aged and irradiated kidneys demonstrate elevated senescence markers, reduced proliferative repair after injury, and produce TGF-β as a part of their SASP to induce fibrosis. Using the senolytic ABT-263 to eliminate senescent proximal tubular epithelial cells improves these parameters (152). A recent study demonstrated renal tubular epithelial cell senescence – evidenced by higher p16^INK4a^, p19, and p21^CIP1^ expression – secondary to chronic ischemia from renal artery stenosis in mice and humans. A dasatinib and quercetin senolytic combination alleviated renal dysfunction in these mice, suggesting a causal relationship between chronic ischemia, cellular senescence, and kidney damage (153). Diabetic nephropathy is the major cause of CKD worldwide and a significant contributor to obesity-related kidney disease. Biopsies of kidneys from patients with type 2 diabetic nephropathy show elevated senescence markers SA-β gal and p16^INK4a^ in proximal tubular epithelial cells, mesangial cells, podocytes, and endothelial cells (154). WT diabetic kidney disease mouse models develop proteinuria and glomerular hypertrophy, and both effects are attenuated in p21^CIP1^-KO mice (155). Dasatinib and quercetin combination also reduce AKI to CKD transition in murine models of cisplatin and radiation-induced kidney injury (156). Senescent markers SA-β gal, p16^INK4A^, and p21^CIP1^ are also elevated in the kidneys of rats and cats with CKD (157, 158). These data show cellular senescence underpins various renal pathologies that lead to CKD, and senolytics could mitigate this progression.

CKD is considered a systemic premature aging phenotype, known as uremia-associated aging (159). Uremic patients can develop age-related conditions, including osteoporosis, sarcopenia, frailty, impaired wound healing, infections, insulin resistance, cognitive dysfunction, hypogonadism, and vascular aging (160–163). Hence, certain uremic toxins expectedly accelerate biological aging hallmarks, including cellular senescence, to precipitate a premature aging phenotype (164). Uremia-induced senescenced was first demonstrated in the aortas of hypertensive rats, where indoxyl sulfate-related cellular senescence was correlated with aortic wall calcification and thickness, a sign of vascular aging (165). Uremia-induced senescence is mediated by oxidative stress and consequent DNA damage response signaling and upregulation of p21^CIP1^ and p53 (166). A recent review by Huang et al. summarized the mechanisms behind CKD-induced senescence (167). The uremic toxins indoxyl sulfate and *p*-cresyl sulfate induce senescence in mesenchymal stem cells, evidenced by elevated p21^CIP1^ expression (168, 169). A normocytic normochromic anemia is common in CKD pateints, and is mainly thought to be due to low erythropoietin production by the kidney. Mas-Oodi et al. recently demonstrated that indoxyl sulfate induced senescence in CD34+ hematopoietic stem cells, thereby arresting their proliferation and reducing erythropoiesis (170). Indoxyl sulfate also induces senescence in renal proximal tubular epithelial cells in CKD through ROS-dependent p53 expression (171). Senescent proximal tubular cells display a proinflammatory and profibrotic protein signature, with elevations in NFκB and TGF-β production, possibly contributing to further declines in renal function (171, 172).

*P*-cresyl sulfate activates NADPH oxidase and induces oxidative stress in mouse 3T3-L1 adipocytes. Exposure to p-cresyl increases TNF-α and IL-6 production by 3T3-L1 adipocytes and increases ATM infiltration, suggesting that this uremic toxin is a mediator of CKD-induced adipose tissue inflammation (172, 173). In agreement with these findings, Koppe et al. reported that administering p-cresyl sulfate to normal mice for 4 weeks triggered lipotoxicity and insulin resistance (174). Although senescence markers were not evaluated in these studies, it is conceivable that the intracellular accumulation of *p*-cresyl sulfate may induce adipocyte senescence, since oxidative stress is the major pathway of adipose tissue senescence. In the context of the adipose-renal axis, these studies suggest that the accumulation of uremic toxins in CKD trigger senescence in adipose tissue, amplifying the inflammaging seen in CKD pateints. Further studies are needed to determine which uremic toxins induce adipose tissue senescence, the mechanisms involved, which subpopulations of cells in adipose tissue are affected, the important SASP components by which uremia-induced senescent adipose tissue exerts pathologic systemic effects, and whether senolytic or other senescent-targeting strategies are effective in ameliorating uremia-induced senescence. Another pathway to consider in the adipose-renal axis in CKD is the gut microbiome. CKD alters the symbiotic relationship between the intestinal microbiome and the body (i.e., gut microbiome dysbiosis), leading to the fermentation of macronutrients and production of various uremic toxins, including indoxyl sulfate, *p*-cresyl sulfate and others. CKD-related impairment of the intestinal epithelial barrier allows for the spillover of these toxins into the bloodstream, which drive systemic oxidative stress and inflammation (175, 176). Whether and how certain dietary modifications lifestyle interventions such as exercise, restore host-enterobiome symbiosis and alleviate senescence in the context of the adipose-renal axis is an important topic for future studies to address.

### 4.3 Senotherapies in obesity and kidney disease

Targeting senescent cells pharmacologically can alleviate numerous age-related diseases. Baker et al. initially demonstrated that depleting senescent cells prevents the development of age-related changes in adipose tissue, skeletal muscle, and eyes of INK-ATTAC transgenic mice (150). Numerous strategies have emerged to deplete senescent cells or mitigate their harmful effects. Briefly, senolytic drugs inhibit SCAPs characteristic of senescent cells, allowing for the selective depletion of senescent cells. Dasatinib, quercetin, and fisetin are the most studied senolytics in preclinical animal models thus far, and numerous clinical trials testing their efficacy in age-related disorders are underway (177). Senomorphic drugs inhibit various SASP components without inducing senescent cell death. Most senomorphics target transcriptional regulators of the SASP, including ATM, p38 MAPK, JAK/STAT, NFκB, and mTOR pathways. Other strategies are also emerging, recently reviewed by Zhang et al. (178, 179). Many preclinical studies have shown senolytics to alleviate aging phenotypes, including cancer, chemotherapy- and radiation-induced premature aging, diabetes, osteoarthritis, neurodegeneration, glaucoma, age-related macular degeneration, idiopathic pulmonary fibrosis, heart failure, and CKD (180).

The impact of senescent cell depletion has also been investigated in obesity and kidney disease. Palmar et al. cleared senescent cells either by senolytic combination dasatinib+quercetin or by selective depletion of p16^INK4a^-expressing cells and observed reduced adipose tissue inflammation and improved glucose tolerance and insulin sensitivity (181). Since DNA damage is the major inducer of senescence in adipose tissue, interventions such as exercise, N-acetylcysteine, and senolytics that reduce oxidative stress in adipose tissue decrease adipose tissue SC burden and attenuate ATM infiltration and adipose tissue inflammation (125). Another study showed that senescent cell clearance in adipose tissue of HFD obese mice by dasatinib+quercetin and navitoclax improved insulin sensitivity and increased plasma adiponectin levels (182). Consistent with higher adiponectin levels, senescent cell clearance anormalizes microalbuminuria and podocyte barrier integrity (181). An HFD increases senescent cell burden in mouse kidneys—detected by p16^INK4a^, p19, and p53 expression and SASP upregulation—linked to renal fibrosis and functional impairment (144). Quercetin administration reduced senescent cell burden in the kidney, attenuated renal fibrosis, increased renal cortical oxygenation, and lowered plasma creatinine levels (144).

These findings suggest that depleting adipose tissue-resident senescent cells by senolytics restores adipogenesis, reduces adipocyte hypertrophy, improves glucose tolerance and insulin sensitivity, reduces macrophage infiltration into adipose tissue, and increases adiponectin secretion.

## 5 Sleep apnea, obesity and CKD

Obstructive sleep apnea (OSA) is a globally prevalent disorder increasing in incidence. OSA is characterized by collapse of the upper airway during sleep, causing arousal with or without oxygen desaturation, leading to sleep fragmentation and daytime sleepiness. Obesity is a strong risk factor for OSA: OSA affects 40% of moderately obese (BMI >30 kg/m^2^) and 90% of severely obese patients (BMI >40 kg/m^2^) (183). A 10% increase in bodyweight increases the Apnea-Hypopnea Index (AHI) by 32%, whereas a 10% decrease in body weight decreases the AHI by 26% (184). Obesity increases pharyngeal collapsibility by reducing upper airway diameter and lung volume, predisposing to collapse and consequent OSA (185).

OSA patients are at a significantly higher risk of stroke, myocardial infarction, arrhythmias, insulin resistance and diabetes, heart failure, pulmonary hypertension, and CKD. The pathogenic hallmark of OSA is chronic intermittent hypoxia (CIH), which exerts direct pathologic effects in multiple organs. Although the kidney receives 25% of the cardiac output, blood flow to the renal medulla is tightly regulated to maintain the interstitial medullary osmotic gradient which facilitates water reabsorption. The renal medulla is, therefore, highly vulnerable to ischemic injury. CIH leads to significant tubulointerstitial damage by increasing oxidative stress and inflammation (186). In agreement with these findings, treatment with lipoic acid, an antioxidant, ameliorates hypoxia-related renal injury by decreasing oxidative stress, renal cell apoptosis, and tubular injury (187). CIH also activates interstitial fibroblasts and induces renal tubular epithelial cells to undergo an epithelial-to-mesenchymal transition by upregulation of HIFs, leading to renal fibrosis (188–191). Nocturnal hypoxia in OSA patients over-activates the sympathetic and renin-angiotensin-aldosterone systems, associated with long-term renal impairment (192–194). These experimental models explain clinical studies showing that OSA contributes to CKD development and progression. For example, a cross-sectional analyzing over 7700 subjects with OSA for CKD revealed that, in additional to traditional CKD risk factors, lower nocturnal oxygen saturation was associated with CKD, with a 2% rise in CKD probability for every 1 unit drop in oxygen saturation (195). Furthermore, studies following OSA patients longitudinally have revealed that nocturnal hypoxia is independently associated with steeper declines in eGFR, cardiovascular mortality, and all-cause mortality (196–200).

Continuous positive airway pressure (CPAP) therapy is the mainstay of treating OSA and resolves CIH. CPAP significantly reduces snoring and daytime sleepiness and improves the quality of life in OSA patients. CPAP significantly decreases renal sympathetic and RAAS activity and blood pressure, improves renal hemodynamics, slows the rate of eGFR decline, and reduces microalbuminuria in patients with severe OSA (201–204). However, data suggest that CPAP may be ineffective at improving renal function in moderate nocturnal hypoxia and men (205, 206). Furthermore, CPAP is ineffective at reducing the incidence of a composite clinical end point of cardiovascular mortality, myocardial infarction, stroke, transient ischemic attack, and heart failure in patients with moderate-to-severe OSA (207, 208). Varying degrees of compliance to treatment among patient groups may partly be responsible for these discrepant findings. Nevertheless, such data highlight the need for elucidating the pathogenesis of OSA-related kidney disease. No pharmacological treatments are currently available for OSA. Identifying mediators of the systemic organ dysfunction caused by OSA may reveal pathways that may be clinically beneficial to target and supplement CPAP therapy to enhance the long-term outcomes of these patients.

In this regard, OSA may alter patients’ adipokine profiles. OSA patients have significantly reduced adiponectin compared to non-OSA patients, regardless of sex, age, or BMI (209, 210). Low serum adiponectin levels are associated with decreased cystatin C urinary excretion in male OSA patients (192). Ding et al. demonstrated that CIH in rats increased oxidative stress and markers of apoptosis in the kidney compared to normal controls (211). Treating CIH rats with adiponectin reduces oxidative stress and renal cell apoptosis (211). Similar results have been observed in cardiomyocytes, neurons, and pulmonary cells (212–214). Improving sleep quality in OSA patients with cardiovascular disease either by CPAP, nocturnal supplemental oxygen, or sleep hygiene education significantly increased serum adiponectin levels and improved glucose tolerance parameters (215). Therefore, low serum adiponectin in OSA may contribute to their higher risk of systemic complications including renal impairment and insulin resistance.

OSA also increases leptin levels with consequent leptin resistance (216). Li et al. concluded that leptin is significantly higher in OSA than non-OSA patients and correlates with a higher AHI (217). Obesity, a frequent comorbidity of OSA, causes hyperleptinemia and leptin resistance. Leptin is key in stabilizing upper airway muscles and stimulating CNS respiratory drive (218–221). Therefore, it is conceivable that leptin resistance may contribute to the higher risk of OSA in obese individuals. Administering leptin intranasally to obese mice alleviates OSA independent of body weight reduction (222). OSA also increases leptin levels and causes leptin resistance through CIH. A recent study reported that CIH for 96 days in rats significantly increased leptin levels (223). Furthermore, while leptin injections into normoxic controls reduced food intake, no such effect was observed in the CIH animals, indicating leptin resistance (223). High levels of leptin drive oxidative stress and chronic inflammation that underlie the long-term cardiovascular complications of OSA (224).

Aging is a significant risk factor for many complications seen in OSA, suggesting that OSA may accelerate aging at the cellular level and precipitate a premature aging phenotype (225–228). Sleep deprivation activates a DNA damage response in peripheral blood mononuclear cells of older adult humans, with consequent increases in p16^INK4a^ expression and elaboration of a SASP (229). Sleep fragmentation also induces senescence in the aorta of adult male C57BL/6J mice (230), possibly related to a pro-oxidant response in the vascular endothelium induced by CIH that accelerates vascular aging (231). Indeed, CIH induces a state of systemic chronic low-grade inflammation through NFκB activation, which can induce senescence (232–234). Lee et al. recently demonstrated CIH in elderly mice to increase lung oxidative stress, inflammation, and fibrosis (235). Many of the pro-inflammatory cytokines measured—such as TNF and IL-6 —are SASP components, although the lungs of these mice were not examined for senescence markers. Polonis et al. reported that OSA-related CIH induces senescence in human preadipocytes—expressing p16^INK4a^, SA-β gal, and γH2AX —through a ROS-dependent pathway (236). The subcutaneous abdominal adipose tissue of OSA patients also demonstrated higher p16^INK4a^ and γH2AX than non-OSA individuals. Importantly, treatment with statins, aspirin and/or a RAS inhibitor significantly reduced senescent cell burden *in vitro* and *in vivo* (236). A recent study by Khan et al. showed that two weeks of CIH increased senescence in the visceral white adipose tissue of C57BL6 male mice through a DNA damage response (146). Adipose tissue senescence was accompanied by increased adipose tissue fibrosis, macrophage infiltration, and inflammation. Furthermore, CIH was associated with the upregulation of pro-fibrotic genes in the myocardium and consequent myocardial fibrosis (146). A major finding of this study was that p53-KO mice (i.e., a defect in a key senescence-inducing pathway) did not develop myocardial fibrosis, and resection of senescent adipose tissue also prevented myocardial fibrosis (146).

In summary, the findings above indicate that CIH, which is a hallmark of OSA, induces senescence in adipose tissue and several other organs, contributing to systemic functional impairment. For future research, it will be important to ascertain whether the available senolytics reduce senescent cell burden in OSA and whether this ameliorates OSA severity and prevents complications. OSA-related senescence has also not been investigated in the kidney but is likely since renal artery stenosis, which, similar to CIH, leads to chronic ischemia that induces senescence in the renal tubular epithelium and causes renal dysfunction (153). It is also important to note that CKD itself can dysregulate sleep and is a risk factor for OSA development and/or progression through a variety of mechanisms (237–240). These observations suggest the existence of a positive feedback loop, whereby OSA, obesity, and CKD all worsen systemic oxidative stress, inflammation, and senescence in multiple organs.

## 6 Conclusion and perspectives

The increasing prevalence of obesity and kidney disease necessitates a better understanding of the mechanisms behind obesity-induced kidney disease. Although weight loss and lifestyle interventions represent the primary modality of treating obesity, peripheral treatments based on normalizing the adipokine profile or reducing senescent burden in obesity could better clinical outcomes in these patients.

We described the most studied adipokines implicated in obesity-induced kidney disease, but a myriad of other adipokines—including resistin, visfatin, angiotensinogen, and lipocalin—have also been studied in this context. Multiple studies have demonstrated the existence of an adipose-renal axis, whereby obesity-derived cytokines and adipokines damage the kidney, and CKD-related metabolic dysregulation accelerates adipose tissue aging and dysfunction. This axis is influenced by senescent cell burden and the presence of sleep apnea, both of which can amplify inflammation in obesity and CKD. Gut microbiome dysbiosis is another pathway to consider in the adipose-renal axis in obesity and CKD (241–243).

How cellular senescence plays into the adipose-renal crosstalk is largely unexplored in both laboratory and clinical studies but is likely since senescent cells accumulate with age, and obesity-related senescence in adipose tissue and other organs is well-established. Investigating senescence in different CKD complications could also reveal novel biomarkers and targets for pharmacologic intervention. Senolytic and senomorphic drugs could have potential clinical practice-changing implications in treating multiple conditions, including obesity and CKD. Still, their efficacy and, more importantly, safety profile remains to be shown in ongoing clinical trials.

## Author contributions

TA, AS, BS, NA, HS, HA, ANS, AR, and ZA participated in the drafting of the manuscript. TA, AS, and ZA conceptualized and revised the manuscript. All authors contributed to the article and approved the submitted version.
